# DPO: Diffuse Pulmonary Ossification – A Diagnostic Challenge

**DOI:** 10.37825/2239-9754.1032

**Published:** 2021-12-23

**Authors:** G. Rea, G. Giacobbe, D. Caroppo, S. Iovine, R. Lieto, M. Bocchino, T. Valente, A. Maglio, A. Vatrella

**Affiliations:** a Dipartimento di Radiologia, Ospedale Monaldi, A.O. dei Colli, Napoli, Italy; b Dipartimento di Medicina Clinica e Sperimentale, F. Magrassi A Lanzara, Seconda Università di Napoli, Italy; c Dipartimento di Anatomia Patologica, A.O. dei Colli, Napoli, Italy; d Dipartimento di Medicina Clinica e Chirurgia, Sezione di Malattie Respiratorie, Università Federico II, Napoli, Italy; e Dipartimento di Medicina e Chirurgia, Sezione Malattie Apparato Respiratorio, Università di Salerno, Salerno, Italy

**Keywords:** Diffuse, Pulmonary, Ossification, Lung, DPO

## Abstract

Diffuse pulmonary ossification (DPO) is a rare condition of DLD (diffuse lung disease) characterized by the presence of metaplastic ectopic bone in the lungs and is less frequent in patients without a clear background of lung diseases. DPO is characterized by very small calcific nodules, often with bone mature located in both lungs and often in peripheral areas of the lungs. Two patterns of DPO have been recognized dendriform and nodular. The dendriform type is less common and is characterized by a coral-like network of bone spiculae along the alveolar septa and is often related to interstitial fibrosis or chronic obstructive lung disease [1]. Recent literature papers indicate that DPO may be a predictor of pulmonary fibrosis, is related to Usual Interstitial Pneumonia (UIP) pattern, and has a higher correlation with Idiopathic Pulmonary Fibrosis (IPF). We present a case of a 41-years-old male with persistent bronchitis who underwent a chest X-ray (CXR) that showed multiple pulmonary small calcified nodules in both lungs. These findings were then defined with a high-resolution computed tomography of the chest (HRCT) that showed multiple small nodules spread in both lungs with a “tree-like pattern”. A lung biopsy was performed to confirm the radiological diagnostic hypothesis of DPO, and further pathological examination showed multifocal areas of mature bone tissue within the lung parenchyma.

## 1. Case report

**Fig. 1:**
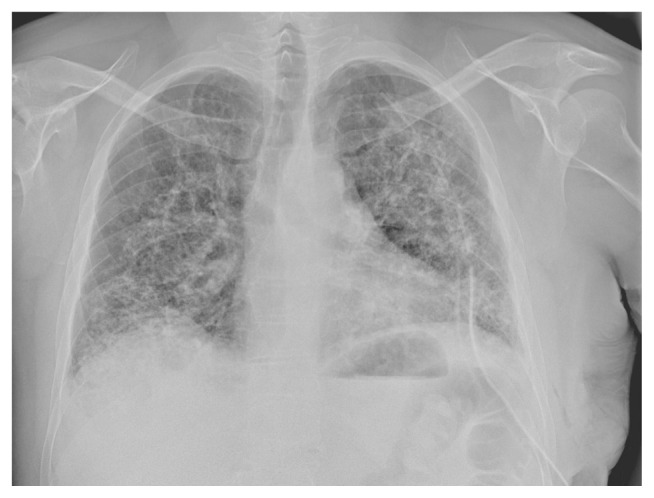
CXR: In both lungs are visible branching and nodular calcific small opacities.

**Fig. 3:**
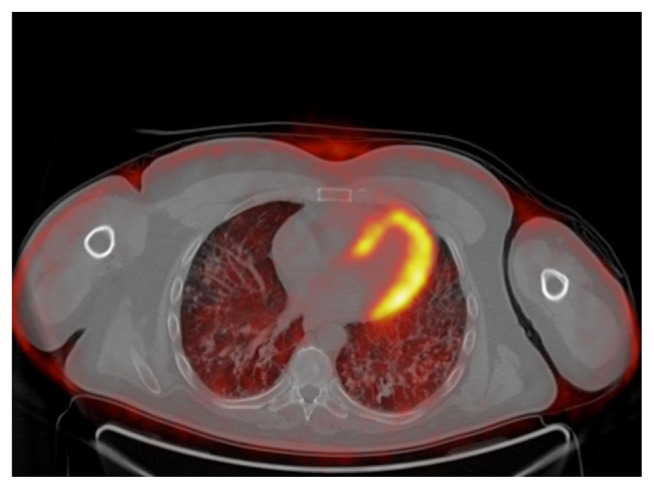
PET/CT examination: not significant increase in metabolic activity in both lungs.

**Fig. 4:**
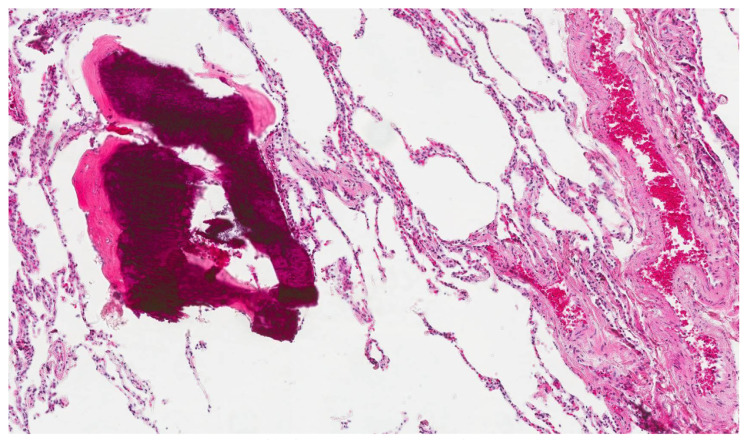
Lung tissue with ossification (left) in the alveolar space (Hematoxylin and eosin stain, 10×).

A 41-years-old male was referred to our hospital for persistent bronchitis. No significant anamnestic findings were evident: no history of smoking, environmental allergen exposure or infective disease, or family history of lung disease or connective tissue disease. Serology almost complete for panel of auto-antibodies was negative (ANA, anti JO-1, MI2, CADM-140,PM/Scl-75/100, topo1/Scl-70, U1-RNP, ANCA, RF, CCP). On physical examination his vital signs were normal. For diagnostic completeness, the patient carried out an ECG trace and an echocardiography which were substantially within the normal limits. Chest examination was negative. Laboratory exams, including complete blood count, electrolyte analyses, and inflammatory markers were normal and also kidney function was normal. The functional tests detected a normal respiratory function but the DLCO/VA was moderately reduced (67% predicted). Arterial Blood gas analysis on room air shows normal blood oxygen and Co2 values in a normal range. Broncho-alveolar lavage (BAL) performed two days after admission, was normal. A chest X-ray (CXR) showed multiple pulmonary small nodules localized in both lungs from the upper fields to the lower fields, with a greater anatomical profusion in the middle fields (Fig. 1). To further characterize these unexpected and non-specific findings, chest CT was performed. CT showed multiple small nodules in both lungs, also along fissures and interlobular septa, with peri-bronchial and peri-lymphatic distribution, associated with thickening of the small airways and the septal interstitium, with a predominantly rosary crown morphology or “tree-like pattern”. The nodules owned the same distribution between the upper and lower lobes in both lungs (Fig. 2a). In mediastinal windows were also visible tiny small calcified nodules expression of DPO. There was no evidence of mediastinal lymph nodes increased in size and/orsigns of air trapping (Fig. 2b). MPR coronal reconstructions showed small nodules in both lungs with a CT appearance of DPO (Fig. 2b). The patient subsequently underwent a PET/CT examination to try to obtain further information capable of increasing diagnostic accuracy and possibly formulating a confident diagnosis that however did not show any significant increase in the metabolic activity in both lungs (Fig. 3). Sarcoidosis was considered among the differential diagnostic hypotheses regarding the incidentally detected CT pattern, but also possible occult exposure pathologies could not be excluded. The use of a second level, interventional investigation was considered mandatory to obtain a precise diagnosis and for this reason, the diagnostic process finally ended with a surgical biopsy (VATS). The histopathological sections lungs with multifocal areas of both intra-parenchymal and subpleural ossifications. Mature bone tissue of variable shape and size was observed inside the alveoli, sometimes conforming to their contours (Fig. 4). The conclusive histological diagnosis was therefore: diffuse pulmonary ossification (DPO). Since he was poorly symptomatic (but with episodes of relapsing bronchitis) the radiological clinical multidisciplinary discussion (MDT) recommended a follow-up in the respiratory clinic and his last pulmonary functional test (24 months after diagnosis) showed a stable lung function. And we decided to carry out a new HRCT only in case of a possible variation of the respiratory symptoms or a variation of the functional data.

## 2. Discussion

Diffuse pulmonary ossification (DPO) is an unusual condition described for the first time by Luschka and colleagues in 1856, defined by the widespread presence of metaplastic bone in the lungs [1,2]. Historically it was considered a post-mortem diagnosis, incidentally detected at autopsy, although in the last few years several cases were recently diagnosed using chest CT or VATS [3–5]. Two types of DPO are described in the literature: nodular and dendriform ossifications [3]. The nodular form is relatively more common and usually occurs in the context of conditions leading to venous congestion, especially mitral valve stenosis. This form is characterized by nodular ossifying masses expanding into the alveolar spaces. Bone marrow is usually absent [6]. The pathogenesis is unclear but the proposed mechanism involves the organization of alveolar exudates that leads to osseous fibroblastic metaplasia, but other mechanisms were investigated: angiogenesis, anoxia, lung injury in fibrosis probably related to the acid environment, finally stimulating fibroblastic proliferation and metaplastic osseous formation [7,18]. Dendriform pulmonary ossification, named because of the dendritic “tree-like” pattern, could be considered either idiopathic more rarely, or secondary. The secondary form usually occurs in association with a variety of underlying disorders such as chronic lung disease, pulmonary amyloidosis, histoplasmosis, sarcoidosis, metastatic disease (osteogenic sarcoma, melanoma, breast cancer), and also IPF and for this reason, DPO finally could be evaluated as a radiological-pathological marker of progressive fibrosis phenotypes [19]. Other conditions in which the dendriform pattern has been described include asbestos exposure, organizing pneumonia (OP), rare earth pneumoconiosis, acute respiratory distress syndrome (ARDS) [3]. DPO is characterized by a coral-like bone network along the interstitium and the alveolar septa; the bony spicules form a contiguous branching pattern that occurs dichotomous at regular intervals with angles of 60–70° every 2 cm [3]. Unlike nodular pulmonary ossification, the ectopic bone tissue of the dendriform type contains marrow elements. DPO is not due to the precipitation of calcium and phosphate in abnormal lung tissue or hypercalcemia but represents a metaplastic osseous formation within the pulmonary interstitium [8]. Although the exact pathogenesis of dendriform pulmonary ossification is still unknown, there is evidence showing a connection between this condition and interstitial fibrosis secondary to inflammation. Has been suggested that the mechanisms involved in dendriform ossification, could be facilitated by post-inflammatory mediators like cytokines and growth factors. In particular, a role for transforming growth factor-beta (TGF-β) was supposed, as this post-inflammatory mediator may stimulate osteoblasts and chondrocytes proliferation [5,9]. TGF-β production is further favored by conditions such as low oxygen tension, low pH, and decreased mechanical compliance, all of which are often found in injured lung tissue [10]. Other mediators such as bone morphogenic protein, interleukin-1 (IL-1), and interleukin-4 (IL-4) have also been reported to induce in vitro ectopic ossification [9].

## 3. Conclusion

Through the literature review (Scopus-PubMed), the analysis of the reported cases, overall, demonstrates that DPO is a prevalent pathological phenomenon most commonly occurs in men in their 40–60s, but in rare cases, it has also been described in younger men and women [11,18]. The male-to-female ratio was about 6:1 with an average age of 60,3 at the time of diagnosis. The lower lobes are most often affected with a predilection for the posterior and lateral basal segments [7,12]. In radiologic literature, DPO has been most often described in association with UIP pattern although in a small minority of cases (DPO was present in only 7% of patients with UIP in the largest series), and the entity is otherwise largely unknown [4,13]. The clinical course is usually indolent, although restrictive physiology and impaired diffusing capacity of the lung for carbon monoxide have been described [14]. Few cases were completely asymptomatic because most of the patients reported respiratory symptoms such as bronchial hyperactivity, cough, and dyspnea. Only in one case, the respiratory symptoms were so severe that they led to a bilateral lung transplant [15]. Pneumothorax was the clinical presentation in rare cases. Co-morbidities with DPO reported in the case series including a history of lung disease as follows: pulmonary fibrosis, COPD, and pneumoconiosis following what previously describe. Previous malignancy was also found in sporadic cases. Furthermore, the occurrence of the same type of dendriform pulmonary ossification in two members of the same family has been observed, which suggests a strong genetic involvement in the progression of the ossification [16]. The DPO was also identified in patients with osteogenesis imperfecta (OI). The pulmonary ossification probably occurred during the repair of alveolar damage caused by decreased collagen type 1 production resulting from OI [17]. In a recent retrospective study Gruden and Coll., have recently identified an association between dendriform pulmonary ossification, in the absence of UIP and chronic aspiration of gastric acid, due to gastro-esophageal reflux disease, obstructive sleep apnea, or a chronic neurologic disorder. Chronic acid aspiration could cause local acidity and hypoxemia that can cause pulmonary fibroblasts to differentiate into osteoblasts [14]. In conclusion, CT scans and more specifically HRCT, are believed to be superior to CXR for correct detection of tiny small calcified nodules and can accurately demonstrate branching lines of calcifications which are characteristics of DPO, with the possibility of carrying out a good differential diagnosis concerning other conditions that can simulate its characteristics, often avoiding the use of surgical biopsy.

Since patients with PDO cannot benefit from specific therapy, the recommendation to treat and control any co-morbidities present is considered necessary, due to the potential fibrosing interstitial evolution linked to this rare disease.

## Figures and Tables

**Fig. 2a:**
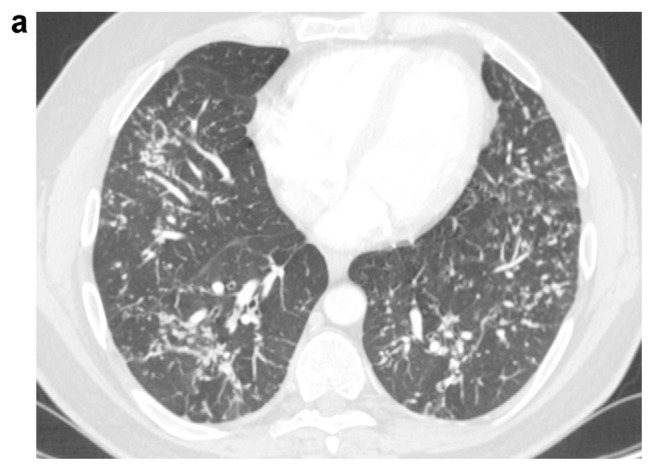
CT scan lung windows (1.25 mm collimation) of lower lung lobes: nodular thickening of contiguous interlobular septa and subpleural tiny nodules in posterior and lateral basilar segments of both lower lobes. Absence of distortion and/or traction bronchiolectasis.

**Fig. 2b:**
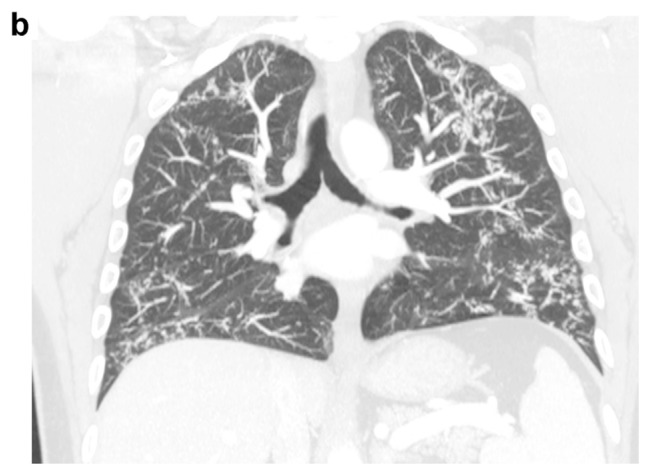
CT scan lung window MPR coronal view: nodular thickening of contiguous interlobular septa and subpleural tiny nodules in both lower lobes. Absence of distortion and/or traction bronchiolectasis.
